# Identifying Biomarkers from Mass Spectrometry Data with Ordinal Outcome

**Published:** 2007-02-05

**Authors:** Deukwoo Kwon, Mahlet G. Tadesse, Naijun Sha, Ruth M. Pfeiffer, Marina Vannucci

**Affiliations:** 1 Division of Cancer Epidemiology and Genetics, National Cancer Institute, Bethesda, MD, U.S.A; 2 Department of Biostatistics & Epidemiology, University of Pennsylvania, Philadelphia, PA, U.S.A; 3 Department of Mathematical Sciences, University of Texas at El Paso, TX, U.S.A; 4 Department of Statistics, Texas A&M University, College Station, TX, U.S.A

**Keywords:** Markov chain Monte Carlo, mass spectrometry, ordinal outcome, variable selection

## Abstract

In recent years, there has been an increased interest in using protein mass spectroscopy to identify molecular markers that discriminate diseased from healthy individuals. Existing methods are tailored towards classifying observations into nominal categories. Sometimes, however, the outcome of interest may be measured on an ordered scale. Ignoring this natural ordering results in some loss of information. In this paper, we propose a Bayesian model for the analysis of mass spectrometry data with ordered outcome. The method provides a unified approach for identifying relevant markers and predicting class membership. This is accomplished by building a stochastic search variable selection method within an ordinal outcome model. We apply the methodology to mass spectrometry data on ovarian cancer cases and healthy individuals. We also utilize wavelet-based techniques to remove noise from the mass spectra prior to analysis. We identify protein markers associated with being healthy, having low grade ovarian cancer, or being a high grade case. For comparison, we repeated the analysis using conventional classification procedures and found improved predictive accuracy with our method.

## Introduction

In recent years, technologic developments have spurred interest in using protein mass spectroscopy to identify molecular markers for discriminating between phenotypic groups [1]. The diagnostic categories often consist of tumor versus normal tissues, different types of malignancies, and subtypes of a specific cancer. Several variable selection methods have been developed to address this problem [2, 3, 4]. These procedures are tailored towards classification into nominal categories. In some cases, however, the outcome of interest may have an ordered scale. Examples of variables with a natural ordering include the stage or grade of a tumor and quantitative clinical factors such as white blood cell counts. Applying methods designed for nominal variables to such problems may not be optimal since the information about the ordering is ignored. Chu et al. [5] have recently proposed a gene selection algorithm based on Gaussian processes to identify expression patterns associated with ordinal phenotypic outcomes in DNA microarray data.

We analyzed surface-enhanced laser desorption/ionization time-of-flight (SELDI-TOF) mass spectrometry data from a proteomic discovery and biomarker validation study for ovarian cancer conducted at the National Cancer Institute [6]. In ovarian cancer, more than two-thirds of cases are detected at an advanced stage, resulting in poor overall five-year survival rates of 10–30% [7]. This is in stark contrast to stage I/IIa patients with 95% five-year survival [7]. Cancer antigen 125 (CA-125) is the most widely used biomarker for ovarian cancer. However, it does not have adequate sensitivity and specificity to be used as a screening tool. Even in conjunction with transvaginal sonography, the positive predictive value of CA-125 is only about 20% [8]. Protein mass spectroscopy has been used previously to identify markers that may improve the diagnostic performance of existing markers for early detection of ovarian cancer [1, 9]. In this paper, we aimed to identify proteomic markers that are related to an ordinal measure of disease severity defined in terms of tumor grade.

Mass spectrometry data are inherently noisy. A pre-processing step is needed before any analysis. Several algorithms have been developed to this end [10, 11, 12]. Here, we adopt a pre-processing approach that uses wavelet techniques to remove noise from the mass spectra. We then propose a Bayesian variable selection method for classifying individuals into ordinal categories and apply the method to the processed mass spectra. In our approach the ordered outcomes are related to the protein levels using a data augmentation approach. The variable selection procedure is built into the model through a latent binary inclusion/exclusion vector. Markov chain Monte Carlo (MCMC) stochastic search techniques are used to update this latent vector and to explore the prohibitively large space of possible predictor combinations. Posterior inference identifies discriminating variables and predicts the ordered group membership of a sample via Bayesian model averaging. This allows us to account for the uncertainty inherent in the model selection process.

We compare our prediction results with those obtained from commonly used classification methods, such as linear discriminant analysis (LDA), quadratic discriminant analysis (QDA), *k*-nearest neighbor (KNN), and support vector machines (SVM). These methods, unlike our Bayesian model, build a multiclass classifier that ignores the natural ordering of the outcome. Moreover, with the exception of SVM, which provides a relevance measure for each variable, these procedures do not perform selection of the discriminating markers.

## Experimental Data

Serum samples collected at the Mayo Clinic between 1980 and 1989 were analyzed by surface-enhanced laser desorption and ionization time-of-flight (SELDI-TOF) mass spectrometry using the CM10 chip type [13]. The ProteinChip Biomarker System (Ciphergen Biosystems) was used for protein expression profiling. Serum samples were analyzed by scientists blinded to disease status at Ciphergen Biosystems. Information on subjects included patient’s age at diagnosis, CA-125 levels, and stage and grade for all the cancer cases. A detailed description of the samples and exclusion criteria can be found in [6]. In this paper, we focus on 50 samples obtained after 1986 whose serum was freeze-thawed a single time. They consist of 10 individuals free of ovarian cancer as well as cases with tumors graded as “well differentiated” (*n* = 5), “moderately differentiated” (*n* = 6), “poorly differentiated” (*n* = 13), and “undifferentiated” (*n* = 16). We defined three ordinal classes based on tumor grade: *Z* = 0 for controls, *Z* = 1 for well or moderately differentiated tumor, and *Z* = 2 for poorly or undifferentiated tumor.

## Methods

### Pre-processing of mass spectrometry profiles

**Figure 1.**
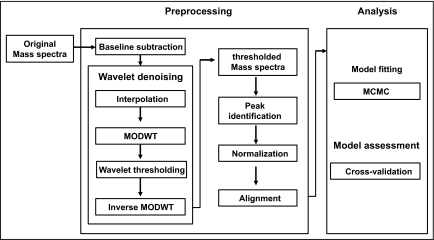
Pre-processing and analysis of mass spectroscopy data.

Protein mass spectra are inherently noisy and require substantial pre-processing before analysis. A mass spectrum can be represented as a curve where the *x*-axis indicates the ratio of a particular molecule’s weight to its electrical charge (*m/z*) and the *y*-axis represents a signal intensity corresponding to the abundance of the molecule in the sample. Most peaks in the spectrum are associated with proteins or peptides and constitute important features. The goal of the analysis is often to identify peaks related to specific outcomes, such as different malignancies or clinical responses. Before proceeding to the data analysis, a number of pre-processing steps, such as removal of baseline and noise, normalization and calibration of samples, are needed. The procedures to perform these steps are still experimental and no standard has yet been established. The pre-processing steps we used are described below and summarized in Figure 1.

#### Baseline correction

This step is required to remove the ion overload and chemical noise that are usually higher at smaller *m/z* values. There is no general solution to this problem because baseline characteristics vary from one experiment to another and each spectrum has to be assessed individually. For the data considered in this paper, the baseline subtraction algorithm implemented in the BioConductor PROcess package (www.bioconductor.org) was used. This function splits a spectrum into a number of exponentially growing regions, calculates the quantiles in each region, and smoothes the results using the loess function.

#### Noise removal by wavelet methods

Wavelets are families of orthonormal bases that can be used to parsimoniously represent functions. Following the seminal work of Donoho and Johnstone [14], wavelet thresholding has successfully been used in various applications to remove noise and recover the true signal intensities[15]. This is accomplished by applying a wavelet transform to the data and mapping wavelet coefficients that fall below a threshold to 0 (hard thresholding) or shrinking all coefficients toward 0 (soft thresholding). One can also opt between a universal or an adaptive thresholding rule. The former applies the same threshold, i.e. identical cut-off value or same amount of shrinkage for all wavelet coefficients, whereas the latter uses a threshold that depends on the resolution level of the wavelet coefficients. An inverse wavelet transform is then applied to the thresholded coefficients leading to a smoothed estimate of the function.

**Figure 2.**
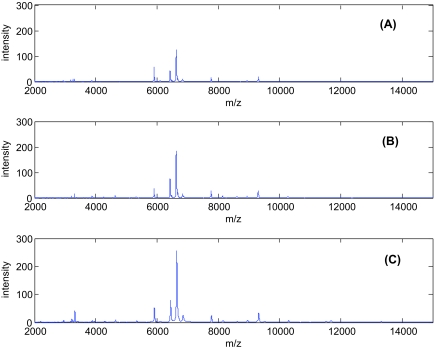
Profiles of three mass spectra from each class.

We discarded *m/z* values lower than 2,000 due to large noise and *m/z* values greater than 15,000 because all the intensities in this range were very low. For the remaining data, we interpolated the mass spectra on a grid of equally spaced *m/z* values with 500,000 equi-spaced points using piecewise cubic splines. We noticed better qualitative denoising with undecimated transforms over standard decimated discrete wavelet transforms (DWT). These transforms do not impose restrictions on the length of the signal and are shift-invariant, i.e. they are not affected by the starting position of the signal. We used the maximum overlap discrete wavelet transforms (MODWT) [16] with Daub(4) along with an adaptive soft thresholding rule. Figure 2 displays spectra plot after baseline correction and noise removal

#### Normalization

When dealing with multiple spectra it is a good practice to remove effects from systematic variation among spectra due to varying amounts of protein or to variation in the detector sensitivity. For this we used a global normalization procedure where mass intensities are scaled by a common factor. For a given peak in a given spectrum we computed the area under this peak, i.e. the sum of all intensities, from all spectra. We then defined the constant factor as the ratio of the area under this peak and the median of areas of all peaks.

#### Peak identification

A crucial step for the identification and quantification of proteins in mass spectra is to find *m/z* values that correspond to peak intensities. We used the peak detection methods implemented in the PROcess library from BioConductor with the default settings. For each spectrum, peaks were identified as *m/z* values with signal intensities satisfying the following criteria: 1) the intensity exceeds a specified threshold value; 2) the intensity exceeds a constant times the median absolute deviation estimate of noise in a given window; 3) the intensity is a local maximum within a given window; 4) the ratio of the area under the peak, i.e. the sum of the intensities within a bandwidth, versus the maximum area among all peaks is greater than a pre-specified constant.

#### Peak alignment

Mass spectra exhibit shifts along the horizontal axis between replicate spectra. In general, the instruments have an accuracy of 0.1 to 0.3% on the *m/z* scale. Thus, detected peaks that have masses within the percentage accuracy are considered identical. We merged peaks that have *m/z* measurements within 0.2% of each other and assigned the new peak the average *m/z* values and the maximum intensity.

### Probit model for ordinal outcomes

Let (*Z,X*) denote the observed data, where *Z_n_* _× 1_ is the vector of ordered categorical outcomes and *X_n × p_* is the matrix of covariates. In our setting, *X* contains the intensities at given *m/z* values. The responses *Z_i_* take one of *J* values, 0, ..., *J* – 1. Each outcome *Z_i_* is associated with a vector (*p_i_*,_0_, ..., *p_i_,_J_* _– 1_), where *p_i,j_* = *P*(*Z_i_* = *j*) is the probability that subject *i* falls in the ordered class *j*. The probabilities *p_i,j_* can be related to the linear predictor *x_i_*β by adopting a data augmentation approach [17]. We assume that there exists a latent continuous random variable *Y_i_*, such that

(1) $$Y_{i}=\alpha +x_{i}\beta +\varepsilon_{i},\varepsilon_{i}∼N\left(0,\sigma^{2}\right),i=1,\ldots ,n,$$

where α is an intercept parameter, β is a *p* × 1 vector of regression coefficients and σ^2^ is set to 1 to make the model identifiable. The correspondence between the observed outcome *Z_i_* and the latent variable *Y_i_* is defined by

(2) $$Z_{i}=j\;\;\text{if}\;\;\delta_{j}\;<Y_{i}\leq \delta_{j+1},j=0,\ldots ,J-1,$$

where the boundaries δ*_j_* are unknown and –∞ = δ_0_ < δ_1_ < ... < δ*_J_* _– 1_ < δ*_J_* = ∞.

### Incorporating variable selection into the model

Without loss of generality, we assume in the sequel that *X* has been centered, so that its columns sum to zero. Thus, rank (*X*) ≤ min(*n* – 1, *p*).

In our application, most of the predictors provide no information about the outcome of interest. In order to identify the informative predictors, we introduce a latent binary inclusion/exclusion vector γ that induces a mixture prior on the regression coefficients. We specify conjugate priors for the intercept $\alpha ∼N(\alpha_{0},h)$ and the regression coefficients of the included variables $\beta_{\gamma }∼N(\beta_{0\gamma },H_{\gamma })$. The simplest form for the prior of γ is to assume its elements to be independently and identically distributed Bernoulli random variables, π(γ) = *w^p_γ_^* (1 – *w*)*^p–p_γ_^*, where *w* is the proportion of variables expected *a priori* to be related to the outcome and *p*_γ_ is the number of included variables. This prior can be relaxed and more uncertainty can be introduced by assuming a further beta prior on *w*.

The method we propose here for variable selection is closely related to the approach presented in Sha et al. [4] for multinomial probit models. In this context, however, the correspondence between *Z_i_* and *Y_i_* uses different boundaries that account for the natural ordering of the outcome. In addition, here *Y* is a vector that follows a truncated normal distribution, whereas in the multi-group classification case *Y* is a matrix and follows a truncated multivariate-t distribution. The resulting Gibbs sampler is therefore computationally less demanding in the ordinal setting.

### Hyperparameter settings

A vague prior can be specified on the intercept parameter α by setting *h* large, so that the value ascribed to the prior mean becomes irrelevant. We set α_0_ = 0 and β_0_ = 0. For a given γ, the prior on β depends on the matrix *H*_γ_. Brown et al. [18] discuss relative merits and drawbacks of different specifications. Here we use *H* = *cI*, which is easier to calibrate. The parameter *c* regulates the amount of shrinkage in the model. In general, we want to avoid very small values of *c* which cause too much regularization and large values that can induce nonlinear shrinkage as a result of Lindley’s paradox [19]. In Sha et al. [4], we provided some guidelines on how to choose this hyperparameter in the context of probit models for classification into nominal groups. We suggest using similar guidelines here. Specifically, we recommend choosing *c* such that the ratio of prior to posterior precision is relatively small. In practice, values of *c* that provide good mixing of the MCMC sampler, with 25–50% distinct visited models are appropriate. For the boundary parameters, we need to impose one constraint to ensure identifiability; without loss of generality we take δ_1_ = 0. For the remaining boundaries, we assign diffuse priors that express no prior belief by setting δ*_j_* to be uniformly distributed on (δ*_j –_* _1_, δ*_j_* _+ 1_).

### Model fitting

The prior beliefs are then updated with information from the data. We perform posterior inference using Markov chain Monte Carlo (MCMC) techniques. The model fitting can be made more efficient by integrating out the parameters α and β. The MCMC sampler starts from a set of arbitrary parameters and the following steps are iterated:

1. Update the latent vector *Y* from its posterior distribution given (γ, δ, *X*, *Z*), which is a truncated normal density under the constraints defined in equation (2)
  (3) $$Y|\left(\gamma ,\delta ,X,Z\right)∼N_{\delta }\left(1_{n}\alpha_{0}+X_{\gamma }\beta_{0\gamma },P_{\gamma }\right),$$
  where *P*_γ_ = *I_n_* + *h*1*_n_*1*_n_*^′^ + *X*_γ_ *H*_γ_ *X*′_γ_, 1*_n_* is an *n* × 1 vector of ones, *I_n_* is an *n* × *n* identity matrix.
2. Update the latent variable selection vector γ from its conditional posterior distribtion
  (4) π(γ|Y,δ,X,Z)∝π(γ) · π(Y|γ,δ,X,Z).
  This is accomplished using a Metropolis algorithm as in Sha et al. [4]. In this approach, the sampler visits a sequence of models that differ successively in one or two variables. At a generic step, a candidate model, γ*^new^*, is generated by randomly choosing among a set of transition moves. These moves consist of adding or deleting a variable by choosing one of the γ*_k_*’s (*k* = 1, ..., *p*) and changing its value, or swapping the status of two variables by choosing independently and at random a 0 and a 1 and exchanging their values. The proposed γ*^new^* is accepted with a probability that depends on the ratio of the relative posterior probabilities of the new vector versus the one visited at the previous iteration.
3. Update the boundary parameters δ*_j_* from their posterior densities given (γ, *X*, *Z*, δ_(_*_–j_*_)_), where δ_(–_*_j_*_)_ is the vector δ without the *j*-th element. These conditional distributions are uniform on the interval [max{max{*Y_i_* *: Z_i_* = *j* – 1},δ*_j_*_–1_}, min{min{*Y_i_* *: Z_i_* = *j*},δ*_j_* _+ 1_}], as described in Albert and Chib [17].

### Posterior inference

The MCMC procedure results in a list of visited variable subsets, γ, as well as sampled δ and *Y* vectors with their corresponding relative posterior probabilities. In order to draw posterior inference, we first need to impute the latent vector *Y*, which can be viewed as missing data. Let *Ŷ* and δ̂ be the estimates obtained by averaging respectively over the sampled *Y* and δ vectors. The normalized conditional probabilities π(γ*|Ŷ*, δ̂, *X*, *Z*), which identify promising variable subsets, can be computed for all distinct vectors γ visited by the MCMC sampler. The marginal posterior probabilities of inclusion for single variables, π(γ*_k_* *=* 1|*Ŷ*, δ̂, *X*, *Z*), *k* = 1, ..., *p*, can also be derived from these posterior probabilities.

Inference on class prediction can be done in various ways. If a further set of observations is available for validation, least squares prediction based on a single “best” model can be computed:

(5) $${\hat{Y}}_{f}=\tilde{\alpha }+{\text{X}}_{f(\gamma )}{\tilde{\beta }}_{\gamma },$$

where γ is the vector with highest posterior probability, *X*_γ_ consists of the covariates selected by γ, α̃ = *Ŷ*, β̃_γ_ = (*X*′_γ_ *X*_γ_ + $H_{\gamma }^{-1}$)^–1^*X*′_γ_*Ŷ*. Alternatively, we can use Bayesian model averaging over a set of *a posteriori* likely models to estimate *Y_f_* :

(6) $${\hat{Y}}_{f}=\sum_{\gamma }(\tilde{\alpha }+{\text{X}}_{f\;(\gamma )}{\tilde{\beta }}_{\gamma })\;\pi \left(\gamma |\hat{Y},\hat{\delta },X,Z\right).$$

The ordered categorical outcomes can then be predicted using the correspondence

(7) $${\hat{Z}}_{f,i}=j\;\;if\;\;{\hat{\delta }}_{j}<{\hat{Y}}_{f,i}\leq {\hat{\delta }}_{j+1}$$

In situations where the sample size is limited, which is typical in genomic and proteomic experiments, dividing the data into a training and a validation set may not be possible. In such cases, one can resort to sampling-based methods for cross-validation prediction [20]. A cross-validation predictive distribution for sample *i* can be calculated using π(γ,*Y*, δ*| X*, *Z*) as importance sampling density for π(γ, *Y*, δ***|*** *X*, *Z*_(_*_–i_*_)_), where *Z*_(–_*_i_*_)_ is the outcome vector *Z* without the *i*-th element:

(8) $$\begin{array}{l} \;P(Z_{i}=j\;|\;X,Z_{\left(-i\right)})\\ =\int_{\gamma }\int_{Y}\int_{\delta }P(Z_{i}=j\;|\;X,Z_{(-i),}\gamma ,Y,\delta )\\ \times \;\;\pi (\gamma ,Y,\delta \;|\;X,Z_{\left(-i\right)})d\delta \;\;dY\;\;d\gamma \\ \propto \frac{1}{M}\;\sum_{t=1}^{M}P(\delta_{j}^{\left(t\right)}<Y_{i}\leq \delta_{j+1}^{\left(t\right)}|X,Z_{\left(-i\right)},\gamma^{\left(t\right)},Y^{\left(t\right)})\\ =\frac{1}{M}\;\sum_{t=1}^{M}\Phi (\delta_{j+1}^{\left(t\right)}-\mu_{Y_{i}}^{\left(t\right)})-\Phi \left(\delta_{j}^{\left(t\right)}-\mu_{Y_{i}}^{\left(t\right)}\right). \end{array}$$

where *t* indexes the MCMC iterations, Φ(.) is the normal cumulative density function, $\mu_{Y_{i}}^{\left(t\right)}=\alpha^{\left(t\right)}+x_{i,\gamma (t)}\beta_{\gamma }^{\left(t\right)}$ with *α^(t)^* = *Ȳ^(t)^*, $\beta_{\gamma }^{\left(t\right)}={\left({X^{\prime }}_{\gamma^{\left(t\right)}}X_{\gamma^{\left(t\right)}}+H_{\gamma }^{-1}\right)}^{-1}{X^{\prime }}_{\gamma^{\left(t\right)}}$. *Y^(t)^* and *x_i_*_γ_*^(t)^* are sample *i*’s mesurements for the variables selected by γ*^(t)^*. The class membership of sample *i* can then be predicted by the mode of the predictive distribution:

(9) $${\hat{Z}}_{i}=\underset{0\leq j\leq J-1}{\text{arg}\text{max}}P(Z_{i}=j\;|\;X,Z_{\left(-i\right)}).$$

## Results

Figure 2 displays the pre-processed mass spectra for three women randomly chosen from each of the three groups. Each spectrum represents the expression profile of peptides defined by their *m/z* values. We note some clear differences between the three curves. We pre-processed the spectra as described in the Methods section. After applying the wavelet thresholding for noise removal, the peak identification and alignment steps resulted in 39 peaks.

**Figure 3.**
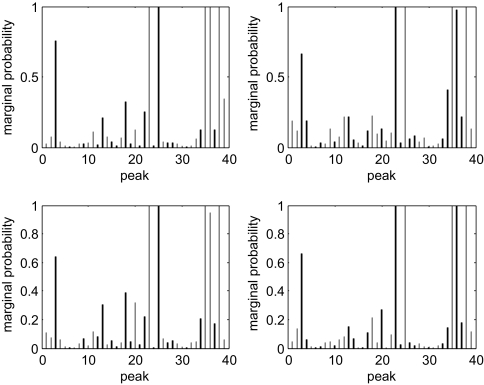
Marginal posterior probabilities of inclusion for single peaks in each of the four MCMC chains.

**Figure 4.**
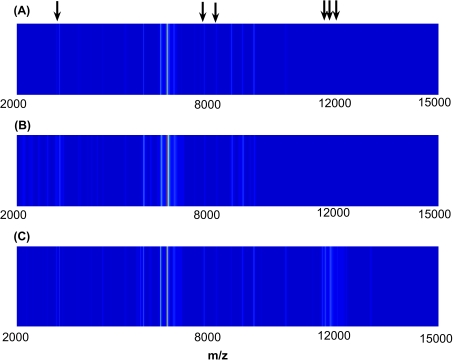
Surface representation of spectra from patients in the three classes. Arrows at the top of the graph indicate peaks selected by our method.

We fitted the ordinal probit model with variable selection to identify protein markers that discriminate among the three groups. We used a Bernoulli prior with 10 variables expected to distinguish the classes. We ran four MCMC chains with widely different starting values for 100,000 iterations each and discarded the first half as burn-in to eliminate dependence on the starting points. We considered several hyperparamater values for the covariance of the regression coefficients, with *c* ranging between 0.1 and 10. Although there was minimal effect on the overall results, we found that smaller values of *c* tended to allocate a couple more samples from ‘low grade’ into ‘control’, and for larger values of *c* a couple more samples from ‘low grade’ were being misclassified as ‘high grade’. Here, we report the results for *c* = 3. Each chain visited about 22,000 distinct models after the burn-in period. The majority of the visited models contained around 10 variables. The marginal probabilities of inclusion for single peaks for each of the four MCMC chains are shown in Figure 3. Indices with high posterior probabilities correspond to important markers that discriminate between the different groups. We note that despite the widely different starting models, similar regions are visited by the different MCMC runs and there is a good concordance among the four plots. We therefore drew posterior inference on the pooled output from the four MCMC chains. We considered variables with large marginal posterior probabilities as well as markers included in the “best” models, i.e. γ vectors with high joint posterior probabilities. The list of selected markers based on marginal probabilities of inclusion greater than 0.1 and based on the best model are reported in Table 1. We note that there is a good agreement between the results. The best model contain seven markers, which are denoted by asterisk characters. Six of these are also selected based on their marginal probabilities of inclusion. Figure 4 displays surface representations of single spectra in each of the three groups for *m/z* values between 2,000 and 15,000. The arrows on top of the graph indicate peaks that appeared in the best model. We note that they clearly distinguish the different groups. For comparison, we performed Kruskal-Wallis analysis of variance on each peak to identify those that are significantly different between the three classes. There were 6 peaks with p-values less than 0.1. Their corresponding *m/z* values (and *p*-values) are: 5,819.138(0.072); 11,427(0.026); 11,514.5(0.0014); 11,673.5(0.001); 11,724.75(0.002), 11,903(0.004). Three of these (underlined values) overlap with the peaks selected by our method.

We used the cross-validation prediction approach described in the Methods section to assess the predictive ability of the selected discriminants. The results are reported in Table 2. We obtained an overall misclassification rate of 19/50 = 0.38. For comparison, we analyzed the data using common classification methods, such as linear discriminant analysis (LDA), quadratic discriminant analysis (QDA), *k*-nearest neighbor (KNN), and support vector machines (SVM), which build multi-class classifiers without taking the natural ordering of the response into account. In addition, except for SVM which gives a relevance measure for each variable, these methods do not provide a selection of the discriminating markers. For QDA, we obtained best results by first applying principal component analysis (PCA) to the data and performing the discriminant analysis on 5 components. For KNN, we considered values of *k* ranging from 2 to 8 and we report the results for k = 3, which gave the lowest overall misclassification rate. We note that all the procedures had higher error rates compared to our method. In particular, our approach performed much better in separating class I and class III, which correspond respectively to disease free and poorly- to non-differentiated samples. As we noted above, the standard classification approaches do not perform variable selection. A common practice in applying these classification methods consists of first running univariate tests to identify significantly different variables. The selected subset of variables is then used in the classification algorithm. We repeated the comparison with the standard classification methods using this two-stage approach. For each of the standard classification procedures, we assessed their cross-validation errors by considering the spectra found to be differentially expressed across the three groups. This was achieved by running an analysis of variance and selecting the spectra with *p*-values less than 0.1 at every leave-one-out prediction [21]—there were 4 to 11 variables selected. This approach resulted in higher misclassification error rates for all the methods compared to their performance based on the whole spectral data.

## Discussion

We have proposed a Bayesian approach for classification problems with ordinal outcomes and high-dimensional predictor data. While MCMC techniques are generally computationally intensive, our method is fairly straightforward. Once we augment the data and introduce latent variables underlying the ordinal outcomes, the problem reduces to variable selection in linear model setting, with the additional requirements of updating the latent continuous variables and their boundaries. We have made our Matlab code for implementing this procedure available at www.stat.tamu.edu/mvannucci/webpages/codes.html.

We have illustrated the performance of our method with an application to mass spectrometry data from an ovarian cancer study. The ordinal outcome groups consisted of a control group and two case groups defined in terms of tumor differentiation. The overall cross-validated prediction accuracy was close to 62%. Not surprisingly, most of the misclassified samples were from the cases with well and moderately differentiated tumors, which would be expected to be difficult to capture. The prediction errors, however, could also be attributed to the relatively long storage time of the samples, which may have laid to degradation of some proteins. Nonetheless, our method identified 11 peaks as possible predictors. Several of those peaks correspond to proteins that have previously been shown to be associated with ovarian cancer. One of the predictive peaks, *m/z* value 3,271 we believe is inter-α tyrpsin inhibitor heavy chain 4 (ITIH4), which has been found to predict ovarian cancer by Zhang et al. [9], Fung et al. [22] and Song et al. [23]. However, our findings are based on small number of samples in each group and need to be confirmed in larger studies. Ordinal outcomes not only occur when dealing with tumor stages, but also in settings where one wishes to associate an environmental exposure with protein levels in serum or urine. For example, in an ongoing study, we are applying our method to mass spectrometry data obtained from urine samples of subjects with low, moderate and high levels of exposure to arsenic in drinking water. The identified markers can subsequently aid in etiologic studies of arsenic exposure and cancer outcomes.

We have also proposed wavelet-based techniques for pre-processing the raw mass spectrometry data. We explored different choices of wavelet basis (Haar wavelets, Daubechies wavelets, least symmetric Daubechies wavelets) and different thresholding rules (hard versus soft and universal versus adaptive). In general, the universal hard threshold removes lots of coefficients and the universal soft threshold tends to attenuate some of the distinctive peaks. The adaptive soft thresholding approach, on the other hand, does a better job at preserving the peaks. We therefore used soft and adaptive wavelet thresholding to remove noise from the spectra. In the future, we plan to investigate alternative approaches, such as block shrinkage methods [24].

## Figures and Tables

**Table 1.** List of selected markers with median intensities for each group.

**median m/z**	**Control**	**Low grade**	**High grade**	**marginal prob.**	
3271	6.3926	3.2527	7.2641	0.4378	*
5743.5976	0.50085	0.49787	1.0655	0.2737	
6540.7	4.2977	3.1079	3.4107	0.3174	
7056.6	2.994	2.8814	2.6191	0.219	
7661.8	2.4026	1.7608	1.4349	1	*
8151.8	5.4292	5.6189	7.312	1	*
11514.5	0.17743	0.19802	0.85362	0.9956	*
11673.5	0.28511	0.31944	1.2318	0.9984	*
11724.752	0.601	0.56101	1.385	0.2497	
11903	0.2833	0.26976	0.73907	0.9998	*
13324.5	1.23	1.1709	1.2205	0.1224	*

**Table 2:** Cross-validated misclassification rates with leave-one-out spectral data used for training classifiers.

**Prediction approach**	**overall error rate**	**Controls**	**Low grade**	**High grade**
MCMC pooled output				
Bayesian prediction	0.38	2/10	8/11	9/29
LDA	0.66	6/10	8/11	19/29
QDA (with PCA)	0.52	3/10	8/11	15/29
KNN (with *k* = 3)	0.48	5/10	8/11	11/29
linear SVM	0.54	2/10	10/11	15/29
nonlinear SVM	0.66	1/10	11/11	21/29
